# Fabrication of Flexible PDMS Films with Micro-Convex Structure for Light Extraction from Organic Light-Emitting Diodes

**DOI:** 10.3390/nano13152216

**Published:** 2023-07-30

**Authors:** Eun-Jeong Bae, Yeon-Sik Kim, Geun-Su Choi, Byeong-Kwon Ju, Dong-hyun Baek, Young-Wook Park

**Affiliations:** 1 Nano and Organic-Electronics Laboratory, SunMoon University, Asan 31460, Republic of Koreacrs4964@korea.ac.kr (G.-S.C.); 2 Display and Nanosystem Laboratory, Department of Electrical Engineering, Korea University, 145, Anam-ro, Seoul 02841, Republic of Korea; n2ur0n@korea.ac.kr; 3 Department of Nano & Semiconductor Engineering, Tech University of Korea, Siheung 15073, Republic of Korea

**Keywords:** organic light-emitting diodes (OLEDs), light extraction, outcoupling, micro-lens array (MLA), micro-convex structure, flexible film, simple process, flexible device, polydimethylsiloxane (PDMS), flexible PDMS film, breath figure, polystyrene

## Abstract

In this study, we demonstrated organic light-emitting diodes (OLEDs) outcoupling with a flexible polydimethylsiloxane (PDMS) film with a micro-convex structure using the breath figure (BF) method. We can easily control the micro-convex pattern by adjusting the concentration of polystyrene and the humidity during the BF process. As process conditions to fabricate the micro-convex structure, polymer concentrations of 10, 20, 40, and 80 mg/mL and 60, 70, and 80% relative humidity were used. To evaluate the optical properties, we analyzed the transmission, diffusion, and electroluminescence with or without the micro-convex structure on the OLEDs. The shape and density of the micro-convex structure are related to its optical properties and outcoupling and we have experimentally demonstrated this. By applying a micro-convex structure, it achieved up to a 42% improvement in the external quantum efficiency compared to bare OLEDs (without any light extraction film). We expect the fabricated flexible light extraction film to be effective for outcoupling and applicable to flexible devices.

## 1. Introduction

Organic light-emitting diodes (OLEDs) have many merits because of their fast response, self-emitting characteristics, low power consumption, and wide color gamut. Especially, OLEDs can be formed with thin film structures, which can be applied to flexible, wearable, or stretchable devices, and can be attractive display devices in wearable and light applications [1,2,3,4,5]. However, although OLEDs have many strong points, it has been a significantly important issue that their external quantum efficiency (EQE) remains as low as ~20% because of the mismatching refractive index of OLED components causing total internal reflection, meaning the light can be trapped as a substrate mode or waveguide mode, surface plasmon polaritons (SPPs) between organic and metallic interfaces [6,7,8]. The light that cannot be extracted is absorbed inside the device and generates heat, thereby degrading device performance. Furthermore, because a high density of current is applied to devices for achieving high luminance, this has an effect on device degradation [9]. To overcome these problems, many studies have been reported on improving the light extraction efficiency of OLEDs. Several approaches have been suggested such as the micro-lens array, surface roughness, buckling patterns, photonic crystals, and antireflection coatings [10,11,12,13,14,15]. Most of the suggested methods required expensive equipment for manufacturing a complicated structure [16,17,18]. Among these methods, the micro-lens array that has been reported has a simple process and can be applied to scalable devices to reduce the total internal reflection loss at the air and substrate interface (substrate mode). Then, many researchers are trying to overcome light extraction using different geometrical structures and various materials [19,20]. A. Kim. et al. (2019) [21] reported an improvement of 23% by fabricating flexible OLEDs on a parylene substrate with a micro-lens array pattern to increase the outcoupling efficiency; Ding. Y. et al. (2020) [22] reported fabricated polydimethylsiloxane (PDMS) films containing the micro-lens array using a replicating process that can increase the brightness of OLEDs by 14%, and Moller, S. et al. (2002) [23] reported that a micro-lens array using a lens diameter of 10 mm increased the EQE of OLEDs by at least 50%. In addition, our previous study (2022) also reported an EQE improvement of 38% by combining nanostructures using a reactive ion etching process on a hemispherical micro-lens array [15], and reported an enhancement in the EQE of 33% using a microsphere-patterned flexible PDMS film [24]. There is room for improvement in the light extraction efficiency enhancement achieved in our previous studies. Furthermore, to increase the efficiency of OLEDs, a cost-effective and less labor-intensive method for fabricating various geometries is needed.

The breath figure (BF) method is an alternative method to fabricate micro-structures because it is a simple and cost-effective method which condenses water droplets in the air on any surface of the substrate by the solvent in dissolved polymers. In the BF method, many parameters affect the shape and dimensions of the formed structure, including the choice of solvent, concentration of polymer solution, atmospheric temperature, and humidity. The essential elements to obtain a good arrangement are: first, the polymer solution has to be coated with a uniform thickness. Second, the substrate should avoid the coalescence of the condensed water droplets.

In this paper, we suggested a fabrication methodology of a flexible light extraction film with various polystyrene (PS) concentrations and humidities. The geometry, size, and distribution of the light extraction structure were evaluated along with the optical properties, and we proved the enhancement in the light extraction efficiency of OLEDs with a micro-lens film through their electroluminescent (EL) characteristics and viewing angle measurements. Finally, we expect that the novel fabrication method based on the BF method can be used to realize the enhanced EQE of OLEDs and other photo devices as a simple and cost-effective methodology.

## 2. Experimental

### 2.1. Fabrication of PS Master Mold and Flexible Micro-Convex Film

**Figure 1:**
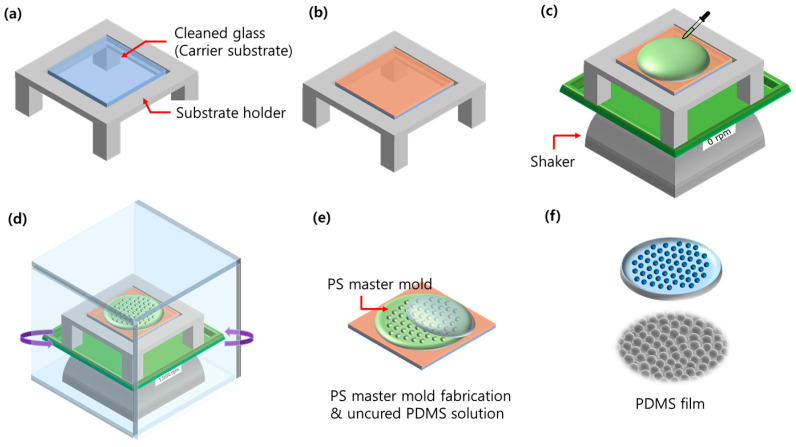
Illustration of the process of obtaining the micro-convex pattern on the flexible PDMS film with the breath figure method and replicating the process. (**a**) glass preparation, (**b**) Attach polyimide film, (**c**) Pour PS solution, (**d**) shaking, (**e**) Pattern transfer from PS master mold to PDMS film, (**f**) micro-convex PDMS film.

The PS solution consisted of PS and dichloromethane (CH_2_Cl_2_), which was used as the solvent. A volume of 4 mL of CH_2_Cl_2_ was put into glass tubes with a pipette and then 40, 80, 160, and 320 mg of PS was put into the glass tubes. This was dissolved with a vortex mixer for 30 min at room temperature (RT). The fabrication process of the PS master mold is shown in Figure 1. (a) The substrate holder was prepared, 35 × 35 mm^2^ of the cleaned glass was prepared as a carrier substrate. (b) The polyimide film was attached to the carrier substrate. (c) A volume of 500 µL of the PS solution was dropped onto the film surface at 60, 70, and 80% relative humidity (RH) using an ultrasonic humidifier. (d) An orbital shaker was used to evaporate the solvent for 5 min and to keep a uniform thickness of the PS master mold. (e) After fabricating the PS master mold, the uncured PDMS was spin-coated on the PS master mold at 1000 rpm, and the uncured PDMS was cured on a hot plate at 80 °C for 20 min. (f) For releasing the hemispherical PDMS film from the PS master mold, the fully cured PDMS film on the PS master mold was dipped in acetone with ultrasonic. Finally, we obtained a micro-convex structure on the PDMS film which was replicated from the PS master mold.

### 2.2. OLED Device Fabrication and Characterization

**Figure 2:**
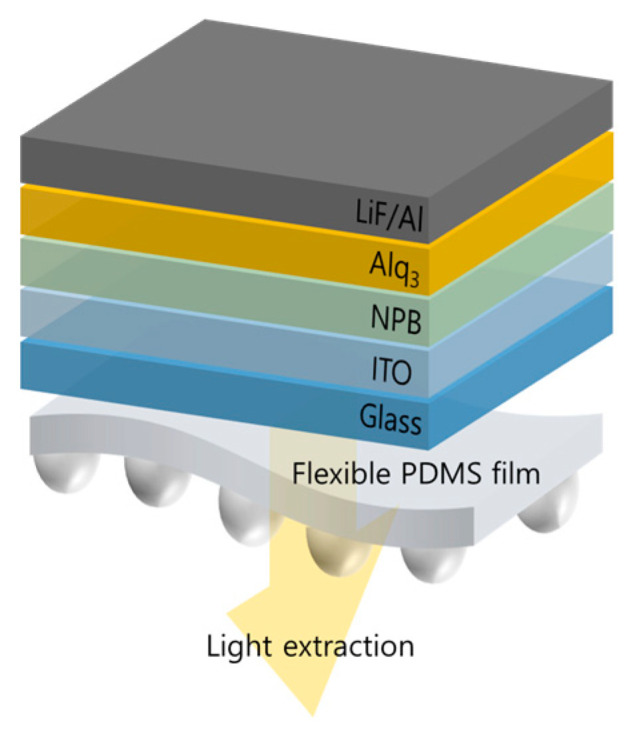
The OLED structure. The flexible PDMS film with micro-convex structure was attached on the outside of the OLEDs.

To fabricate the OLEDs, we cleaned a 1-inch glass substrate, then deposited 185 nm of indium tin oxide (ITO) on the glass substrate by using ultrasonic with acetone, methanol, and deionized water (DI water. Then, the substrate was dried in an oven for 1 h. To define the emission area of the OLEDs, we performed a photolithography process using a photoresist (AZ GXR 601, AZ Electronic Materials CO., Ltd., Darmstadt, Germany). After that, the surface of the substrate was treated with UV–ozone for 30 min (UVC-300, Omniscience, Gyeonggi-do, Republic of Korea), and oxygen plasma was treated for 160 s with 60 W (CUTE, Femto Science Co., Gyeonggi-do, Republic of Korea) for removing the impurities and reducing the driving voltage, adjusting the work function of ITO. The OLED structure used 185 nm of ITO for the anode, 60 nm of N,N′-Bis(naphthalen-1-yl)-N, N′-bis(phenyl)benzidine (NPB) as the hole transport layer, 60 nm of Tris(8-hydroxyquinoline) aluminum (Alq_3_) as the emission and electron transport layer, 1 nm of lithium fluoride (LiF) as the electron injection layer, and 150 nm of aluminum (Al) as the cathode. All of the organics and metals were deposited with a thermal evaporator under ~10^−7^ Torr of process vacuum and deposition rates of ~1.0 Å/s and ~3.0 Å/s, respectively. To control the thickness of the thin film, a 6 MHz quartz crystal microbalance (Phillip Technologies, Greenville, SC, USA) and thin film deposition controller (IQM-233, INFICON, Bad Ragaz, Switzerland) were used in the deposition process. The flexible film of the micro-convex structure was attached to the glass substrate of the fabricated OLEDs. The structure of the OLEDs is shown in Figure 2.

We observed the surface of the flexible PDMS film with field emission scanning electron microscopy (SEM; Helio G4, Thermofisher, Waltham, MA, USA) to observe the geometrical structure. The Image J software was used to analyze the size distribution in order to characterize the micro-convex structure on the flexible PDMS film. To analyze the optical properties of the film, we measured the perpendicular transmittance and total transmittance with a UV–vis spectrometer (HP 8453, Agilent Technologies Inc., Santa Clara, CA, USA and UV 2600i, SHIMADZU, Kyoto, Japan). The diffuse transmittance and haze were calculated from the measured data of total transmittance and perpendicular transmittance. To evaluate the light extraction of the fabricated micro-convex structure, we compared the light extraction of the attached flexible PDMS film on a glass substrate. The light extraction was compared before and after the PDMS film was applied to the outside of the emission OLEDs. The EL characteristics, including the viewing angle, were measured in a dark box with a spectroradiometer (CS-2000, Konica Minolta Co., Ltd., Tokyo, Japan) and source meter (2410, Keithley, Tektronix, Beaverton, OR, USA) under ~10^−3^ Torr of low vacuum using a vacuum chamber. The viewing angle was measured at 5° intervals from 0 to 70° using an automated rotation stage. The EQE and power efficiency (PE) were recalculated using the viewing angle characteristic.

## 3. Result and Discussion

### 3.1. Micro-Convex Structure According to the PS Concentration Control

**Figure 3:**
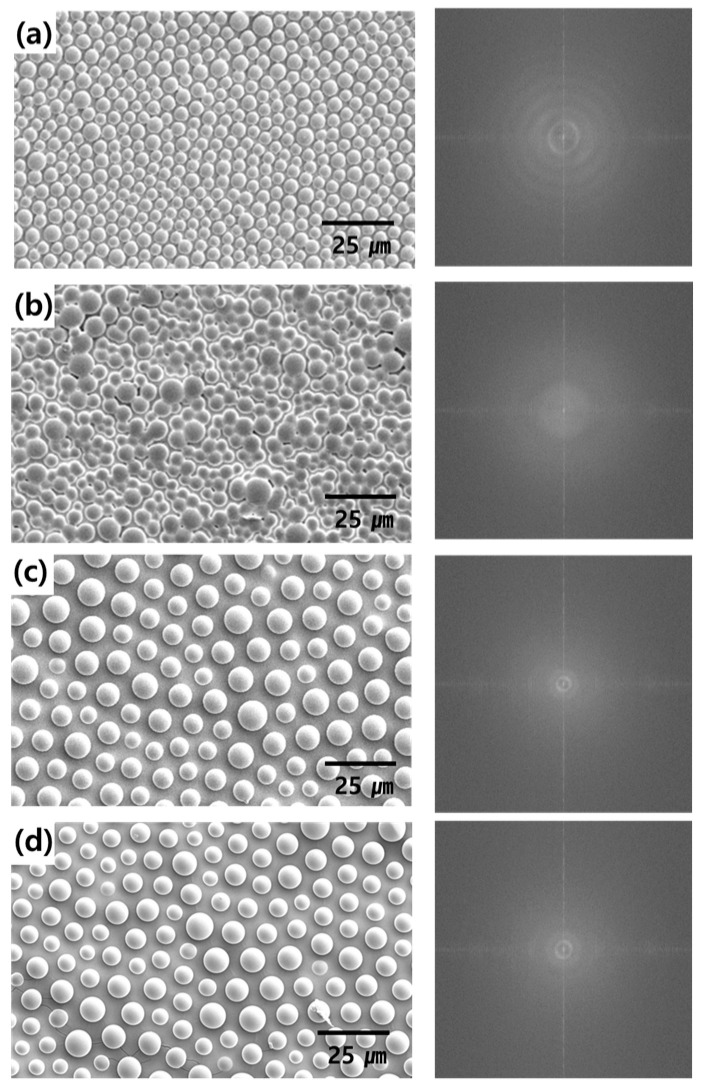
SEM images of surface (top view). The flexible PDMS film at 70% RH made with (**a**) 10 mg/mL, (**b**) 20 mg/mL, (**c**) 40 mg/mL, and (**d**) 80 mg/mL PS concentration (scale bar: 25 μm, right image: fast Fourier transform patterns of each image).

Figure 3 shows an SEM image of the flexible PDMS film with a micro-convex pattern. At this time, the humidity was fixed at 70% during the BF process and the concentration of PS was adjusted to 10, 20, 40, and 80 mg/mL. The size and density of the micro-convex structure depend on the concentration of PS. As the PS concentration increases, the density decreases, and the size of the convex pattern increases. Figure 3a shows a uniform and highly dense structure, and Figure 3b shows a less ordered arrangement compared to Figure 3a. In Figure 3c,d, as the PS concentration increases to 40 mg/mL or more, there is no pattern change and saturation is achieved. Fast Fourier transforms of the patterns on the PDMS films allow for an evaluation of the structures’ factors. The fast Fourier transforms of the 10, 40, and 80 mg/mL PS samples produced symmetrical rings, having various center to center distances that indicated the micro-convex pattern had a random distribution. The 20 mg/mL of PS concentration result confirmed the more disordered structure.

**Figure 4:**
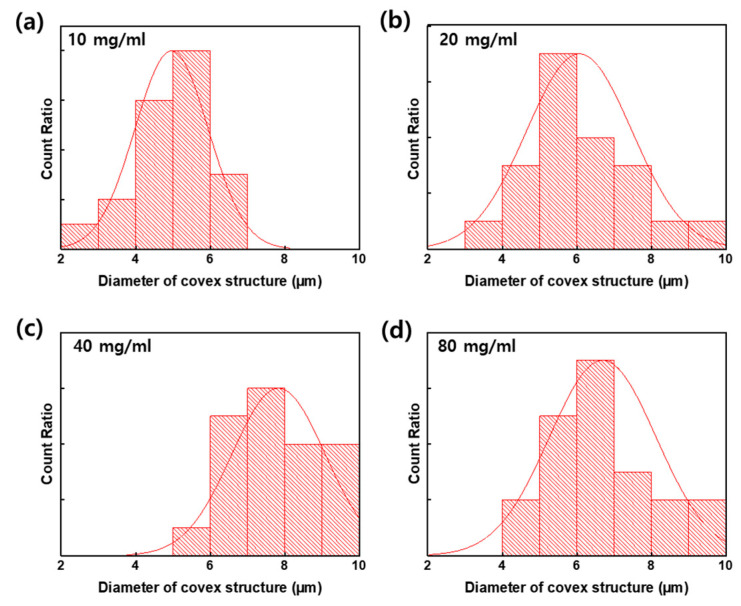
The ratio of size distribution of the micro-convex structure on the flexible PDMS film according to the PS concentrations: (**a**) 10 mg/mL, (**b**) 20 mg/mL, (**c**) 40 mg/mL, and (**d**) 80 mg/mL.

Figure 4 shows the size distribution of the micro-convex structure on the flexible PDMS film. We analyzed the diameter of the convex structure based on Figure 3 using the Image J software. Structures with a diameter of 5–6, 5–6, 7–8, and 6–7 μm were the most counted in Figure 4a–d, respectively. The average diameters of the micro-convex structures calculated from the Gaussian distributions were 5.0, 6.0, 8.0, and 7.0 µm at PS concentrations of 10, 20, 40, and 80 mg/mL, respectively. The diameter of the convex structure with a PS concentration of 20 mg/mL is most diversely distributed, between 3~10 μm. In the SEM image of Figure 3, the PS concentrations of 40 and 80 mg/mL seemed to be saturated, but there was a slight difference in the diameters of the convex structures.

**Figure 5:**
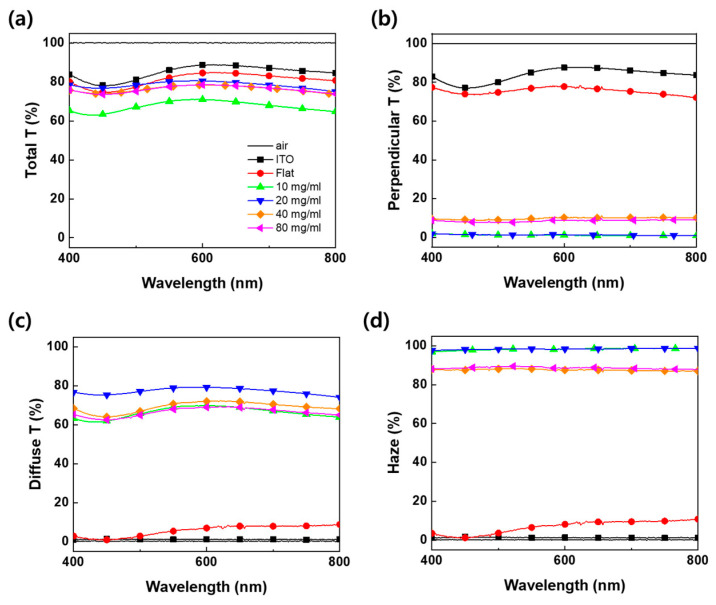
The optical characteristics of flexible PDMS film according to the PS concentration: (**a**) total transmittance, (**b**) perpendicular transmittance, (**c**) diffuse transmittance, and (**d**) haze.

The optical properties of the fabricated flexible film were measured using a UV–visible spectrometer (Figure 5). The degree of light scattering is directly related to the light extraction efficiency. So, the total transmittance, perpendicular transmittance, diffuse transmittance, and haze were analyzed. The diffuse transmittance and haze were calculated using Equations (1) and (2).

Diffuse Transmittance = Total Transmittance − Perpendicular Transmittance(1)

Haze = (Diffuse Transmittance/Total Transmittance) × 100% (2)

All optical properties were evaluated for air, ITO glass without any film attached, transparent flat PDMS film without light extraction pattern, and the fabricated light extraction film according to the PS concentration. The ITO glass shows a total transmittance of more than 80% in the range 400~800 nm (visible light area), and because the light does not scatter, most of the light is transmitted in the straight direction. It shows a diffuse transmittance and haze close to 0%. The flat PDMS film without micro-convex pattern hardly diffuses light, but the incident light sequentially passes through the ITO glass (*n* = 1.5)/patterned PDMS film (*n* = 1.4)/air (*n* = 1.0) interface, the total transmittance, and perpendicular transmittance show a slight loss due to the difference in refractive index. All of the patterned PDMS films exhibit lower total transmittance than the ITO glass and flat PDMS films. Due to the micro-convex structure, the propagating light is hardly transmitted in the straight direction but is transmitted in the lateral direction, showing high haze due to scattering. The film with a PS concentration of 20 mg/mL shows the highest total transmittance and diffuse transmittance. This structure is expected to have the best light scattering due to the most disordered pattern among the PS concentration conditions. On the other hand, the film with a PS concentration of 10 mg/mL showed the lowest total transmittance and it was almost similar to the diffuse transmittance. Since this pattern has the highest density of structures and the smallest size, it appears that light is scattered and trapped inward before being extracted outward, reducing the total transmittance. The films with PS concentrations of 40 and 80 mg/mL show almost the same characteristics, with differences in transmittance and haze within about 2% due to their saturated structures. Table 1 summarizes the optical properties of the flexible PDMS films.

**Figure 6:**
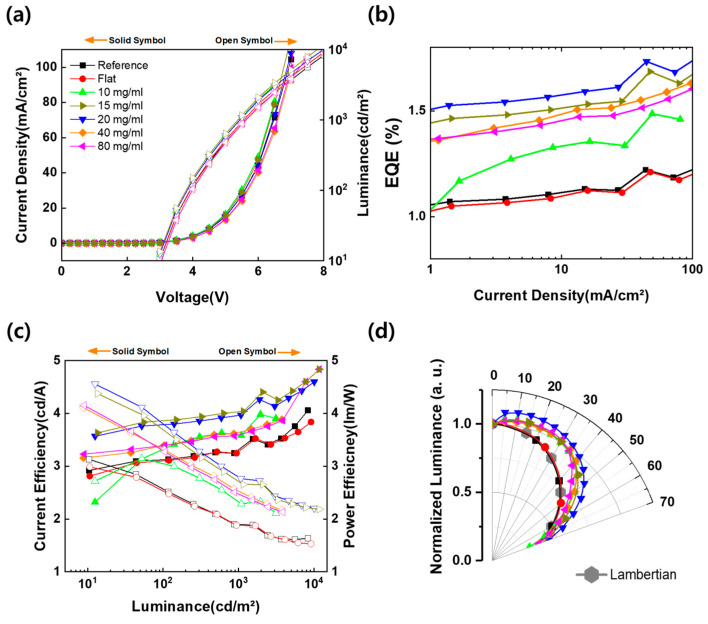
EL characteristics of OLED devices without or with micro-convex structure according to the PS concentration. (**a**) Current density–voltage–luminance (J-V-L) curve (the solid symbols are the current density, on the left Y-axis; the open symbols are the luminance, on the right Y-axis). (**b**) Current density–EQE (J-EQE) curve. (**c**) Current efficiency and power efficiency versus luminance (CE-L-PE) (the solid symbols are the CE, on the left Y-axis; the open symbols are the PE, on the right Y-axis). (**d**) Angular intensity distribution.

To evaluate the light extraction performance of the fabricated micro-convex structures according to the PS concentration, the flexible film was attached to the glass substrate of a green fluorescent OLED with ITO (185 nm)/NPB (60 nm)/Alq_3_ (60 nm)/LiF (1 nm)/Al (150 nm). Figure 6a shows the J-V-L characteristics of OLEDs without or with a micro-convex structure. The J-V curves of all the OLEDs are almost identical, which means that the light extraction film does not affect the electrical characteristics because it is attached to the outside of the OLED devices. Figure 6b shows the EQE curve as a function of current density. At 20 mA/cm^2^, the EQE of the reference without the patterned film and with the flat PDMS film attached is 1.13%. The flat PDMS shows the same value of EQE as the reference because there is no light extraction structure. On the other hand, the EQEs of the 10, 15, 20, 40, and 80 mg/mL PS concentration films are 1.34, 1.54, 1.60, 1.50, and 1.48%, respectively. All of the devices with a light extraction structure were improved compared to the reference. Among them, the EQE of the 20 mg/mL PS film showed the highest value; it was improved by 42% compared to the reference. This result is related to the transmittance characteristics according to the structure and density of the micro-convex structure. It is estimated that when the direction of the light is changed, more light is extracted due to the high diffuse transmittance [25]. In Figure 6c, the current density and power efficiency show the same trend as the EQE characteristics. Figure 6d shows the normalized angular distribution of luminance measured from 0 to 70° (interval angle is 5°) using a rotation stage. The emission profiles of the reference and flat PDMS are almost similar to the Lambertian. All of the devices with the micro-convex pattern show a broader angular distribution than the Lambertian, while the radiative area has the same trend as the EQE characteristics. Furthermore, the light scattered by the convex structure is extracted more in the lateral direction than in the straight direction. The EQE characteristics of all of the devices are summarized in Table 2.

### 3.2. Micro-Convex Structure According to Humidity Control in BF Process

**Figure 7:**
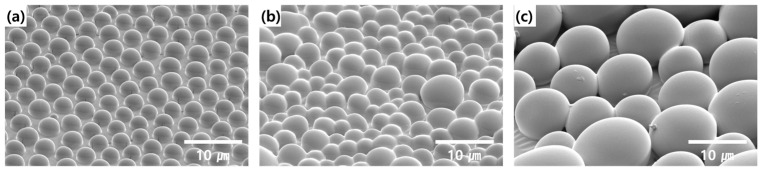
SEM image tilted 45° according to the humidity control in the BF process: (**a**) 60% RH, (**b**) 70% RH, and (**c**) 80% RH. (Scale bar: 10 μm).

In this section, the PS concentration of 20 mg/mL was fixed and the light extraction efficiency according to the humidity change was additionally analyzed. Except for the effect of the PS concentration, the effect of humidity on the diameter and shape of the pattern on the PDMS film was evaluated. Well-ordered patterns, as seen in Figure 7a, were formed at 60% RH, through there being a lower volume of condensed water at the polymer and water interface. However, increasing the volume of condensed water, i.e., increasing the RH, produces increases in the diameter and a randomly ordered distribution, as shown in Figure 7. CH_2_Cl_2_ is known as an amphiphilic solvent that helps the formation of ordered structures in accordance with humidity. Consequently, it is possible to control the diameter, and the ordered or randomly distributed structures depending on the PS solution and humidity.

**Figure 8:**
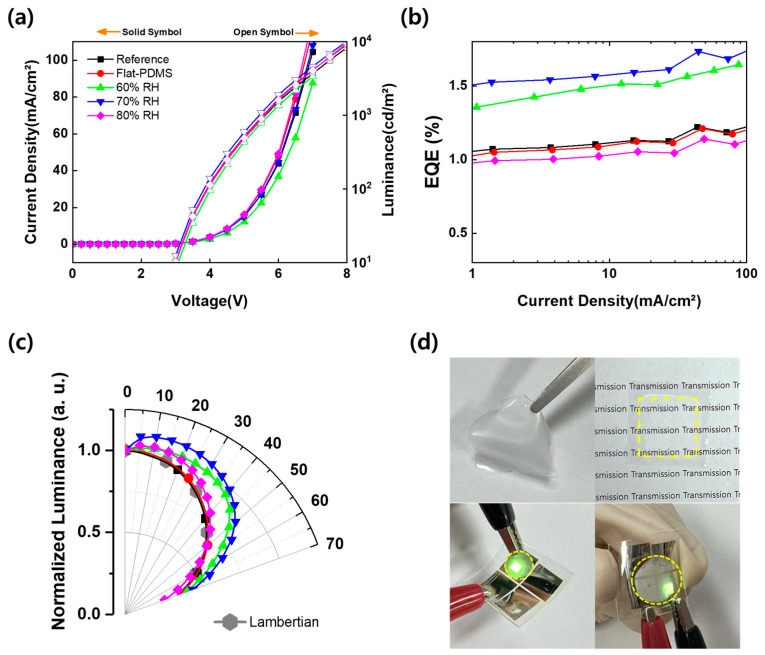
EL characteristics of OLEDs without or with micro-convex structure according to the process humidity. (**a**) Current density–voltage–luminance (J-V-L) curve (the solid symbols are the current density, on the left Y-axis; the open symbols are the luminance, on the right Y-axis), (**b**) current density–EQE (J-EQE) curve, (**c**) angular intensity distribution, and (**d**) photographs of flexible film and flexible OLEDs with the PDMS film with micro-convex structure on 7 V.

We also evaluated the different patterns fabricated through humidity control with OLEDs having the same structure as in the previous section. Figure 8a shows the J-V-L curve. As described above, the film shows the same J-V curve in all conditions without any effect on the driving characteristics of the device. Figure 8b shows the J-EQE characteristics. At a current density of 20 mA/cm^2^, the EQE with relative humidity fixed at 60, 70, and 80% RH is 1.51%, 1.60%, and 1.05%. The highest efficiency was shown at 70% RH. This result is the same as the results described in Section 3.1. The device with 80% RH shows a rather lower efficiency, −7.96% lower than the reference. Figure 8c shows the viewing angle characteristics. The viewing angle shows the same trend as the EQE. The devices with humidities of 60 and 70% RH tended to increase the EL intensity as the angle increased. However, the intensity of the 80% RH device was highest in the straight direction and equivalent to the Lambertian emission as the angle increased. As shown in Figure 8d, the fabricated film shows that it is flexible and can be bent without cracking when attached to flexible devices. Table 3 presents a summary of the EQE characteristics with humidity control in the BF process.

In this study, the control of humidity and PS concentration during the BF process is directly related to the size and density of the light extraction structure, and the pattern can be easily controlled. We experimentally analyzed the density and size of the convex structure when controlling the PS concentration during the BF process, and the size and shape change with humidity control. The EL characteristics of the OLEDs with the flexible light extraction film showed a dependence on the arrangement of the micro-convex structure, and the highest EQE enhancement of 42% was shown at a humidity of 70% RH and a PS concentration of 20 mg/mL. The most effective pattern for light extraction was the most disordered among the patterns investigated in this study, and the diameter distribution of the micro-convex structure was diverse. As a result, the light-through pattern with a PS concentration of 20 mg/mL and humidity of 70% RH was scattered to the lateral direction rather than a straight direction, and this structure showed the highest diffuse transmittance and improved viewing angle characteristics.

## 4. Conclusions

We demonstrated that light extraction flexible film could be fabricated by using a simple and cost-effective breath figure (BF) method. Furthermore, the suggested method can modify the optical properties with the diameter and shape of the micro-convex pattern based on controlling the PS concentration and humidity. As the polymer concentration increased, the diameter of the micro-convex pattern increased, and the spacing between the patterns tended to broaden. In addition, the shape and diameter of the pattern could be controlled by adjusting the humidity during the fabrication of the polymer master mold. During the BF process, PS concentrations in the range of 10 to 80 mg/mL and 60 to 80% RH were applied. The PDMS film made at 70% RH and a PS concentration of 20 mg/mL showed the highest diffuse transmittance of 77.89% at 518 nm. Furthermore, the EQE improved by 42% compared to the reference device (without any light extraction film). This micro-convex structure, analyzed by fast Fourier transforms and the Image J software, shows a disordered pattern and the diameter distribution was diverse, between 3 and 10 μm. So, most of the light was scattered in a lateral direction, and the viewing angle characteristics showed an increased EL intensity according to the angle.

Finally, we can easily fabricate the micro-convex pattern on the light extraction layer by the proposed method and it enhanced the light extraction efficiency of OLEDs. We demonstrated that this study effectively extracted light from the substrate and air interface (substrate mode). We expect that the fabricated flexible film will be widely used because it is effective in light extraction and can be applied to flexible, wearable, stretchable devices or light sources at the same time.

## Figures and Tables

**Table 1:** Summary of the films’ transmittance and haze at 518 nm. (The emission peak wavelength of a green fluorescence OLED in this study).

PDMS Film (PS Concentration)	Transmittance (%) at 518 nm			
	Total	Perpendicular	Diffuse	Haze
Flat PDMS	79.44	75.59	3.85	4.85
10 mg/mL	68.20	1.15	67.05	98.32
20 mg/mL	79.20	1.31	77.89	98.35
40 mg/mL	76.51	9.13	68.55	88.25
80 mg/mL	76.24	7.80	66.24	89.46

**Table 2:** Summary of the EQE and enhancement ratios at 20 mA/cm^2^ with fabricated OLEDs without or with PDMS film, according to the PS concentration.

Device with Film (PS Concentration)	EQE (%)	
	At 20 mA/cm^2^	Enhancement Ratio (%)
Reference	1.13	0%
Reference OLEDs + flat PDMS	1.13	0%
Reference OLEDs + 10 mg/mL	1.35	+19%
Reference OLEDs + 15 mg/mL	1.54	+36%
Reference OLEDs + 20 mg/mL	1.60	+42%
Reference OLEDs + 40 mg/mL	1.50	+37%
Reference OLEDs + 80 mg/mL	1.47	+34%

**Table 3:** The EQE characteristics of OLEDs according to the humidity control during the process.

Device (RH)	EQE (%)	
	At 20 mA/cm^2^	Enhancement Ratio (%)
Reference	1.13	0%
Reference OLEDs + flat PDMS	1.13	0%
Reference OLEDs + 60% RH film	1.52	+34.51%
Reference OLEDs + 70% RH film	1.60	+41.60%
Reference OLEDs + 80% RH film	1.04	−7.96%

## Data Availability

Not applicable.
